# The Rab3 GTPase cycle modulates cardiomyocyte exocytosis and atrial natriuretic peptide release

**DOI:** 10.1016/j.bpj.2025.03.013

**Published:** 2025-03-20

**Authors:** Kobina Essandoh, Arasakumar Subramani, Sribharat Koripella, Matthew J. Brody

**Affiliations:** 1 Department of Pharmacology, University of Michigan, Ann Arbor, Michigan; 2 Department of Internal Medicine, University of Michigan, Ann Arbor, Michigan

## Abstract

Natriuretic peptides are produced predominantly by atrial cardiomyocytes in response to cardiovascular stress and attenuate cardiac maladaptation by reducing blood pressure, blood volume, and cardiac workload primarily through activation of natriuretic peptide receptors in the kidney and vasculature. However, mechanisms underlying cardiomyocyte exocytosis and natriuretic peptide secretion remain poorly defined. Manipulation of Rab3a GTPase activity by Rab3gap1 was recently found to modulate atrial natriuretic peptide (ANP) release by cardiomyocytes. Here, we examined upstream signaling mechanisms and the role of the Rab3a GTPase cycle in exocytosis and ANP secretion by cardiomyocytes. Pharmacological inhibition of the heterotrimeric G protein subunit G*⍺*q suppressed ANP secretion at baseline and prevented GTP loading of Rab3a and ANP release in neonatal rat cardiomyocytes in response to phenylephrine (PE). Similar to agonist-induced activation of ANP secretion, genetic overexpression of a constitutively active, GTP-loaded Rab3a mutant (Q81L) in neonatal rat cardiomyocytes resulted in enhanced intracellular distribution of Rab3a at endomembranes peripheral to the Golgi and promotion of ANP release, indicating that enhancement of Rab3a activity is sufficient to elicit ANP secretion by cardiomyocytes. Collectively, these data indicate G*⍺*q signaling downstream of receptor activation and Rab3a-regulated secretory pathway activity and exocytosis facilitate ANP release by cardiomyocytes that could potentially be harnessed to antagonize hypertension and adverse cardiac remodeling in cardiovascular disease.

## Significance

Atrial natriuretic peptide (ANP) is released by cardiomyocytes as an adaptive mechanism to reduce cardiac workload by reducing blood volume and blood pressure through activation of receptors in target organs including the vasculature and kidney, yet molecular control of cardiomyocyte exocytosis and ANP secretion remains elusive. The Rab3 GTPase cycle was found to be instrumental to induction of cardiomyocyte secretory pathway activity and ANP release. Moreover, GTP loading of Rab3a and ANP secretion downstream of receptor activation were dependent on G*⍺*q, providing a potential mechanistic link from agonist-mediated activation of G*⍺*q-coupled receptors to induction of cardiomyocyte secretory pathway flux and exocytosis by activation of the Rab3a cycle.

## Introduction

Activation of G*⍺*q-coupled receptors, such as the angiotensin-II type 1 (AT1R) and *⍺*1-adrenergic receptors, elicits enhanced atrial natriuretic peptide (ANP) synthesis and release by cardiomyocytes (1,2) as well as increased activation (GTP loading) and total levels of Rab3a (3), a small GTPase with established roles in exocytosis (4,5,6). However, mechanisms linking upstream sensing and initiation of ANP secretion by sarcolemmal receptors and downstream induction of secretory pathway activity and exocytosis in cardiomyocytes that ultimately yields ANP release into the extracellular space have not been well established. Circulating natriuretic peptide levels are used as biomarkers of heart failure pathogenesis because of their increased release by the heart with more disease severity (1,7,8) but also serve a compensatory mechanism to lower cardiac workload through their potent vasodilatory and diuretic activity (1,7,9,10). The natriuretic peptides ANP and B-type natriuretic peptide (BNP), following co-secretional cleavage to their bioactive peptide hormones, bind and activate the transmembrane guanylyl cyclase receptor, natriuretic peptide receptor A (NPR-A), in the vasculature, kidney, and heart, resulting in intracellular cGMP production that promotes vasodilation, natriuresis, and diuresis and antagonizes cardiac hypertrophy (1,7,9,11,12). Natriuretic peptide signaling has been successfully targeted clinically with sacubitril, an inhibitor of the natriuretic peptide-degrading metalloprotease neprilysin, which, when used in combination with the angiotensin receptor blocker, valsartan, improves outcomes in heart failure with reduced ejection fraction through simultaneous elevation of circulating natriuretic peptide levels and antagonism of the renin-angiotensin-aldosterone system, respectively (13,14,15,16,17). Despite the clinical relevance to heart failure and hypertension, the molecular mechanisms governing natriuretic peptide secretion, and exocytosis in cardiomyocytes more generally, remain largely undefined.

Rab3 is a small GTPase that has instrumental roles in regulating exocytosis by facilitating fusion of secretory vesicles with the plasma membrane (5,18,19). As such, Rab3 has indispensable functions in promoting neurotransmitter release (6,20) and perhaps more prominently in directing secretion of neurohormones such as brain-derived neurotrophic factor (BDNF) by neurons (5). Notably, the Rab3 GTPase cycle is essential for exocytosis in neurons as loss of Rab3 GTPase activating protein 1 (Rab3gap1), the catalytic subunit of the Rab3gap enzyme dedicated to hydrolyzing GTP on Rab3, results in a defect in calcium-stimulated glutamate release (6). We recently found that palmitoylation of Rab3gap1 in cardiomyocytes impaired GTPase cycling on Rab3 resulting in sustained steady-state levels of active Rab3a-GTP and inhibition of ANP secretion, whereas acceleration of the Rab3a cycle by overexpression of Rab3gap1 enhanced ANP release (3,21). Collectively, these studies suggest neurons and cardiomyocytes perform successive rounds of Rab3 GTPase cycling to deliver sufficient secretory vesicles to the plasma membrane for release of physiologic levels of hormones, peptides, and neurotransmitters required for endocrine signaling and neurotransmission.

Here, we examined the upstream signaling mechanisms stimulating Rab3 activity, exocytosis, and ANP processing and release and the role of the Rab3 cycle in cardiomyocyte secretory pathway activity and ANP secretion. Rab3 GTP loading and ANP secretion in response to phenylephrine (PE) were dependent on G*⍺*q, suggesting secretory pathway activity, exocytosis, and ANP release in response to agonists of G*⍺*q-coupled receptors may be triggered by Rab3a GTPase activity. Expression of a constitutively active, GTP-loaded mutant of Rab3a increased its stability and localization to peripheral endomembranes/vesicles and promoted ANP secretion and proteolytic processing, whereas a dominant-negative, GDP-loaded Rab3a mutant attenuated ANP release and co-secretional cleavage. Collectively, these data support a role for active Rab3a and its effectors in promoting secretory vesicle trafficking, docking, and/or fusion with the sarcolemma to facilitate cardiomyocyte exocytosis and ANP secretion.

## Materials and methods

### Primary neonatal rat ventricular cardiomyocyte isolation and cell culture

Primary rat neonatal cardiomyocytes were isolated from 1- to 3-day-old Sprague-Dawley rat pup hearts as described previously using the Neonatal Rat Cardiomyocyte Isolation System (Worthington Chemical) (3,22) and cultured and transduced with adenoviruses exactly as previously described in detail (3). All animal procedures were approved by the Institutional Animal Care and Use Committee at the University of Michigan (protocol #PRO00010778) and were in accordance with the Guide for the Care and Use of Laboratory Animals (NIH). Cells were fixed for imaging or lysates and/or secreted medium harvested for biochemical assays as described below.

### Plasmids and adenoviral constructs

GFP-Rab3a (Addgene #49542) (23) or GFP-Rab3a-Q81L (Addgene #49582, gift from Marci Scidmore) cDNAs were subcloned into the pacAd5-CMV-K-N-pA vector (Cell Biolabs) for adenoviral production exactly as described (3) and β-galactosidase (βGal) adenovirus (3,22) was used as a control for transduction.

### Rab3-GTP pull-down and ANP secretion assays

Following plating and adenoviral transduction, neonatal rat ventricular cardiomyocytes (NRCMs) were cultured in serum-free DMEM and harvested 3 or 24 h later to assess Rab3a GTP loading or intracellular and secreted ANP levels by western blotting. For studies with the G*⍺*q inhibitor, NRCMs were pretreated with or without FR900359 (Cayman Chemical, catalog #33666, 1 μM, dissolved in H_2_O) for 1 h and then treated with phenylephrine (10 μM, PE, Sigma #P6126) or phosphate-buffered saline (PBS) in the presence or absence of FR900359 for 3 or 24 h before harvesting cell lysates and conditioned medium for immunoblotting. Conditioned medium was collected and concentrated in Amicon Ultra Centrifugal Filters (10 kDa MWCO, #UFC8010, Millipore) and processed for western blotting as described (3,22). For Rab3a activation assays, NRCMs transduced with adenovirus to express GFP-Rab3a were pretreated with FR900359 (1 μM) for 1 h followed by treatment with PE (10 μM) or PBS for 3 or 24 h with or without FR900359, followed by harvesting for Rab3a-GTP pull-down assays using the GST-Rim1^1−200^ fusion protein using cellular lysates prepared and processed exactly as described in detail previously (3,24).

### qPCR

Total RNA was extracted from cells using the RNEasy mini kit (Qiagen, #74106) according to manufacturer’s protocol. cDNA was synthesized using the High-Capacity cDNA Reverse Transcription Kit (Applied Biosystems, #4368814) followed by qPCR reactions with Power SYBR Green (Applied Biosystems, #4367659) and data acquisition on a QuantStudio 7 Flex (Applied Biosystems) as described previously (3,25). Primer sequences for *Nppa* and *Gapdh* are published (3) and primers for *Rab3a* were For 5′- CAGTGCAGGACTGGTCCAC-3′ and Rev 5′- CCTCGTTCTGAGGACACCAC-3’. Gene expression levels were normalized to *Gapdh* mRNA levels as a housekeeping gene and quantified using the delta-delta Ct method.

### Immunoblotting

Cells were washed two times in cold PBS and intracellular lysates were harvested in radioimmunoprecipitation assay (RIPA) buffer (50 mM Tris-HCl pH 7.4, 1% Triton X-100, 1% sodium deoxycholate, 1 mM EDTA, 0.1% SDS), or in magnesium lysis buffer (25 mM HEPES, pH 7.5, 150 mM NaCl, 1% Ipegal CA630, 10 mM MgCl_2_, 1 mM EDTA, 2% glycerol; Millipore, #20-168) in the case of Rab3-GTP pull-down assays, supplemented with protease inhibitors (1 mM AEBSF, 10 mg/mL leupeptin, and 10 mg/mL aprotinin). RIPA lysates were sonicated (Branson Sonifier 450) and all lysates were clarified by centrifugation. Conditioned medium was collected and concentrated as described in detail (3). Samples were boiled in Laemmli buffer and electrophoresed by standard SDS-PAGE (Bio-Rad) and protein gels stained with SimplyBlue SafeStain (Invitrogen, #LC6060) to assess loading of conditioned medium samples or transferred to PVDF membranes (Millipore, #IPFL00010) for western blotting with the following primary antibodies followed by incubation with IRDye secondary antibodies (1:5000) and imaging and quantification on an Odyssey CLx (LiCor Biosciences) as described previously (26,27): GFP (Novus, NB600-597, 1:1000), Gapdh (Fitzgerald, 10RG109A, 1:50,000), or ANP (Millipore, AB5490,1:500).

### Immunocytochemistry and microscopy

Cells were washed two times in cold PBS before fixation in 4% paraformaldehyde (Electron Microscopy Sciences), washed in PBS for 15 min at room temperature, and immunostained with GM130 (BD Biosciences, #610822, 1:200) or ANP (BMA Biomedicals, T-4009, 1:100) primary antibodies followed by Alex Fluor 647 or 548 secondary antibodies (Thermo Fisher Scientific), respectively, exactly as described previously (3,28). Cells were mounted and nuclei stained with ProLong Gold with 4′,6-diamidino-2-phenylindole (DAPI, Invitrogen, #P36931) followed by image acquisition on a Zeiss LSM 880 microscope in scanning confocal mode with Zen imaging software (Zeiss) at the following excitation/emission wavelengths: DAPI (blue) 405/435 nm, GFP (green) 488/528 nm, red 561/597 nm, and far-red 633/702 nm. ANP immunofluorescence was pseudocolored magenta for clarity of presentation in Figs. 2 *D* and 5 *D*. 40× images containing approximately 8–12 NRCMs per field were acquired with eight images analyzed from each of three experiments conducted on independent NRCM isolations to quantify Manders coefficients for colocalization (29) using ImageJ (NIH) and the JACoP (Just Another Colocalization) plugin (https://imagej.net/ij/plugins/track/jacop2.html) (30).

### Statistical analyses

Statistical analyses and significance testing were performed with GraphPad Prism version 10.2.3 with statistical testing performed as described in the figure legends. Data in histograms are presented as mean ± standard deviation (SD). *p* < 0.05 was considered statistically significant.

## Results

### Gαq inhibition diminishes Rab3a Activation and ANP release

**Figure 1:**
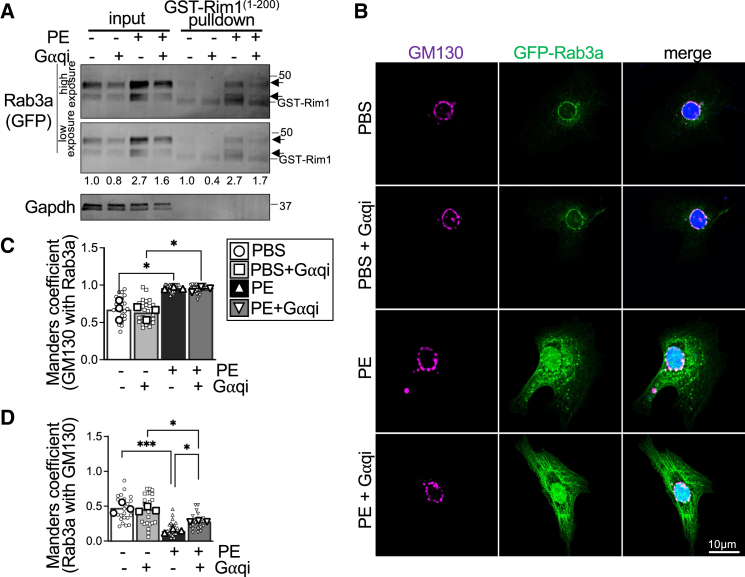
G*⍺*q-dependent activation of Rab3a in cardiomyocytes. (*A*) Rab3a-GTP pull-down assays followed by immunoblotting for input Rab3a protein levels and Rab3a-GTP levels (GST-Rim1^1−200^ pull-down) in neonatal rat cardiomyocytes (NRCMs) transduced with adenovirus to express GFP-Rab3a and treated with PBS or phenylephrine (PE) for 24 h with or without FR900359 (G*⍺*qi, 1 μM). The higher-molecular-weight anti-GFP immunoreactive band migrating around 50 kDa was used for quantification of GFP-Rab3a, although the lower-molecular-weight band reliably shows a similar relative pattern. Total input GFP-Rab3a protein was normalized to Gapdh levels and GFP-Rab3a-GTP levels were normalized to total GFP-Rab3a protein levels in the input. Representative immunoblots are shown from *n* = 4 independent NRCM isolations. Also see Fig. S1. (*B*) Representative images of NRCMs transduced with adenovirus for GFP-Rab3a and treated with PBS or PE for 24 h with or without FR900359 (G*⍺*qi, 1 μM). Cells were immunostained for endogenous GM130 (magenta). Nuclei were stained blue with DAPI. Scale bar, 10 μm. Quantification of Mander’s correlation coefficients for (*C*) the fraction of the Golgi marker GM130 colocalized with GFP-Rab3a and (*D*) the fraction of GFP-Rab3a colocalized with GM130. *n* = 3 independent experiments with 70–100 cells analyzed for each experiment. Individual data points for Manders coefficients obtained from each image are shown, and the mean value for each independent experiment used for statistical testing is depicted by the larger symbols. ^∗^*p* < 0.05, ^∗∗∗^*p* < 0.001, two-way ANOVA with Tukey’s multiple-comparisons test.

Secretagogues such as endothelin-1, angiotensin-II, and PE bind to their respective G protein-coupled receptors (GPCRs) resulting in activation of the heterotrimeric G protein subunit, Gαq, and are *bona fide* activators of biosynthesis and secretion of natriuretic peptides by cardiomyocytes (1,2). Cardiomyocytes treated with PE have elevated Rab3a GTP loading and total Rab3a levels (3). To further investigate the possible involvement of Gαq in activation of Rab3a, we utilized FR900359 (G*⍺*qi), a selective pharmacological inhibitor of Gαq, which has been demonstrated to abrogate PE-induced arterial vasoconstriction in mice (31). To elucidate whether Gαq inhibition modulates Rab3a activity, we subjected cardiomyocyte lysates to pull-down assays employing a GST fusion protein (GST-Rim^1-200^) containing the Rab3 interaction domain of the Rab3 effector, Rab3 interacting molecule 1 (Rim1), to affinity purify active, GTP-bound Rab3a (3,6,19,32). Cotreatment with G*⍺*qi and PE resulted in a similar induction of total Rab3a protein levels compared to cells treated with PE alone after 24 h (Figs. 1 *A* and S1). Although total Rab3a levels were still enhanced in the PE- and G*⍺*qi-treated myocytes, Gαq inhibition did attenuate GTP loading of Rab3a (Figs. 1 *A* and S1). Immunofluorescent imaging showed GFP-Rab3a localization to endomembranes that predominantly colocalized with the Golgi marker GM130 in unstimulated cardiomyocytes but a substantial increase in total GFP-Rab3a protein levels both at the Golgi and a significant increase in localization to vesicles and endomembranes peripheral to the cardiomyocyte nucleus and Golgi (that do not overlap with GM130) in response to PE treatment (Fig. 1 *A*–*D*). These data suggest treatment with the secretagogue and Gαq-coupled receptor agonist PE induces GTP loading, stabilization, and movement of Rab3a from the Golgi to peripheral vesicles or endomembranes. Although Gαq inhibition did not significantly reduce the induction of Rab3a protein levels in response to PE (Figs. 1 *A*–*C* and S1), quantitative analyses revealed that it did partially restore the PE-induced reduction in the portion of Rab3a localized at the Golgi (Fig. 1 *B* and *D*).

**Figure 2:**
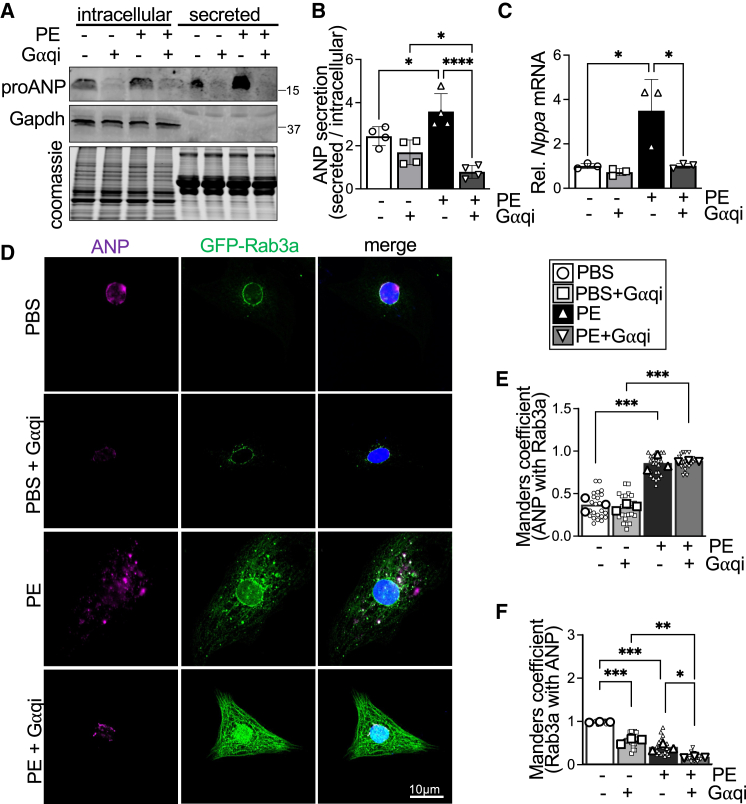
Secretion of ANP is G*⍺*q-dependent. (*A*) Representative immunoblotting for intracellular ANP and secreted ANP in culture medium from NRCMs treated with PBS or PE for 24 h with or without FR900359 (G*⍺*qi, 1 μM). Coomassie staining was used as loading control for secreted protein, and Gapdh levels were used as loading control for intracellular protein. *n* = 4 independent experiments. Quantification of (*B*) the ratio of secreted:intracellular proANP and (*C*) *Nppa* (ANP) mRNA levels in NRCMs treated with or without PE and/or G*⍺*qi. Histograms depict mean ± standard deviation. (*D*) Representative images of NRCMs transduced with adenoviruses for GFP-Rab3a and treated with PBS or PE for 24 h with or without FR900359 (G*⍺*qi, 1 μM). Cells were immunostained for endogenous ANP (magenta). Nuclei were stained blue with DAPI. Scale bar, 10 μm. Quantification of Manders correlation coefficients for (*E*) the fraction of endogenous ANP colocalized with GFP-Rab3a and (*F*) the fraction of GFP-Rab3a colocalized with endogenous ANP. *n* = 3 independent experiments with 70–100 cells analyzed for each experiment. Individual data points for Manders coefficients obtained from each image are shown, and the mean value for each independent experiment used for statistical testing is depicted by the larger symbols. ^∗^*p* < 0.05, ^∗∗∗^*p* < 0.001, ^∗∗∗∗^*p* < 0.001, two-way ANOVA with Tukey’s multiple-comparisons test.

To determine whether pharmacological inhibition of Gαq modulates ANP secretion, we treated cardiomyocytes with PE for 24 h with or without the Gαq inhibitor, collected the NRCM culture supernatant and harvested cell lysates to immunoblot for secreted and intracellular ANP, respectively. As expected, immunoblot analyses revealed that PE treatment substantially elevated intracellular and secreted ANP levels (Fig. 2 *A* and *B*). Most notably, G*⍺*q inhibition completely abrogated ANP secretion at baseline and in response to PE (Fig. 2 *A* and *B*). G*⍺*q inhibitor treatment also reduced intracellular ANP levels at baseline and in response to PE (Fig. 2 *A*) and prevented the induction of *Nppa* mRNA levels in response to 24 h of PE treatment (Fig. 2 *C*), consistent with the established antihypertrophic effect of G*⍺*q signaling blockade in cardiac myocytes (Figs. 1 *B* and 2 *D*) (33,34). Immunocytochemistry revealed PBS-treated control cardiomyocytes showed primarily perinuclear localization of ANP, whereas PE stimulation increased localization of ANP in a punctate pattern at the cardiomyocyte periphery that largely colocalized with Rab3a (Fig. 2 *D* and *E*), suggesting movement of ANP in part to Rab3a-positive post-Golgi vesicles or endomembranes. Consistent with immunoblotting results (Fig. 2 *A*), G*⍺*q inhibition reduced intracellular distribution of ANP protein that was found largely in a Golgi-like perinuclear endomembrane pattern at baseline with increased distribution in a punctate pattern at peripheral endomembranes and vesicles in PE-stimulated NRCMs (Fig. 2 *D* and *F*). Indeed, G*⍺*q inhibitor treatment resulted in a reduction of intracellular ANP protein levels both at baseline and in response to PE (Fig. 2 *A* and *D*) and reduced the portion of Rab3a colocalized with endogenous ANP in response to PE treatment (Fig. 2 *D* and *F*). These data suggest a role for Gαq in activating and potentially localizing Rab3a to post-Golgi vesicles containing ANP and demonstrate Gαq signaling provokes PE-stimulated elevation of Rab3a activity and ANP secretion.

**Figure 3:**
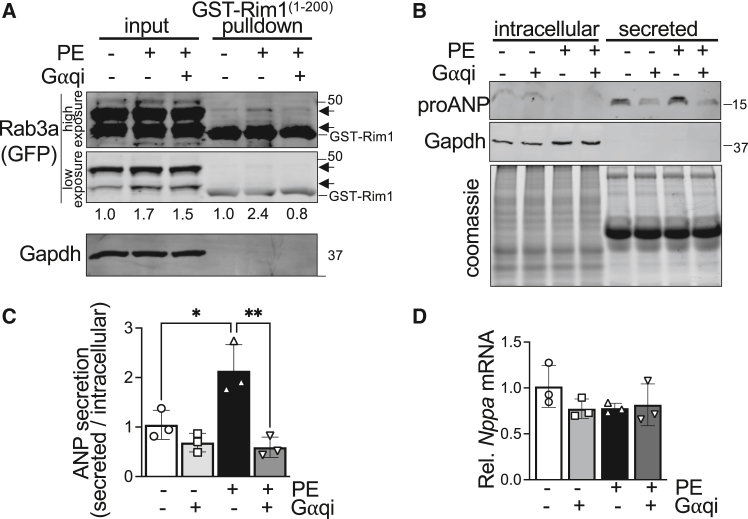
Acute G*⍺*q-dependent activation of Rab3a and ANP secretion. (*A*) Rab3a-GTP pull-down assays followed by immunoblotting for input Rab3a protein levels and Rab3a-GTP levels (GST-Rim1^1−200^ pull-down) in neonatal rat cardiomyocytes (NRCMs) transduced with adenovirus to express GFP-Rab3a and treated with PBS or PE for 3 h with or without FR900359 (G*⍺*qi, 1 μM). The higher-molecular-weight anti-GFP immunoreactive band migrating around 50 kDa was used for quantification of GFP-Rab3a, although the lower-molecular-weight band reliably shows a similar relative pattern. Total input GFP-Rab3a protein was normalized to Gapdh levels and GFP-Rab3a-GTP levels were normalized to total GFP-Rab3a protein levels in the input. Representative immunoblots are shown from *n* = 3 independent NRCM isolations. Also see Fig. S2. (*B*) Representative immunoblotting for intracellular ANP and secreted ANP in culture medium from NRCMs treated with PBS or PE for 3 h with or without FR900359 (G*⍺*qi, 1 μM). Coomassie staining was used as loading control for secreted protein and Gapdh levels were used as loading control for intracellular protein. *n* = 3 independent NRCM isolations. Quantification of (*C*) the ratio of secreted:intracellular proANP and (*D*) *Nppa* (ANP) mRNA levels in NRCMs treated with or without PE and/or G*⍺*qi for 3 h. Histograms depict mean ± standard deviation. ∗*p* < 0.05, ∗∗*p* < 0.01, two-way ANOVA with Tukey's multiple-comparisons test.

Given the multifactorial impacts of Gαq inhibition on ANP biosynthesis and secretion that all collectively contribute to the profound impairment of basal and stimulated ANP secretion (Fig. 2 *A*–*C*), we examined the influence of Gαq in Rab3a activation and ANP release in response to acute PE stimulation. Notably, after just 3 h of PE treatment, Rab3a activation is significantly elevated and this effect is mitigated by inhibition of Gαq (Figs. 3 *A* and S2). Induction of GFP-Rab3a protein levels was observed in NRCMs with 3 h of PE treatment and this effect was not affected by treatment with the Gαq inhibitor (Figs. 3 *A* and S2), indicating Gαq is required for Rab3a GTP loading but not stabilization of Rab3a protein levels downstream of Gαq-coupled receptor activation. Next, we evaluated ANP secretion in NRCMs acutely stimulated with PE in the presence or absence of the Gαq inhibitor and observed a substantial and significant impairment of PE-stimulated ANP secretion as well as a trend toward reduced basal ANP release with Gαq inhibition (Fig. 3 *B* and *C*). Notably, these profound effects of Gαq on ANP secretion in response to acute PE treatment occur in the absence of alterations in *Nppa* mRNA levels (Fig. 3 *D*), indicating Gαq plays roles in PE-stimulated ANP secretion and cardiomyocyte exocytosis that are associated with Gαq-dependent GTP loading of Rab3a (Figs. 3 *A* and S2) but independent of Gαq effects on ANP biosynthesis (Fig. 2 *A*–*C*).

### Constitutive Rab3a activity is sufficient to stimulate ANP secretion

**Figure 4:**
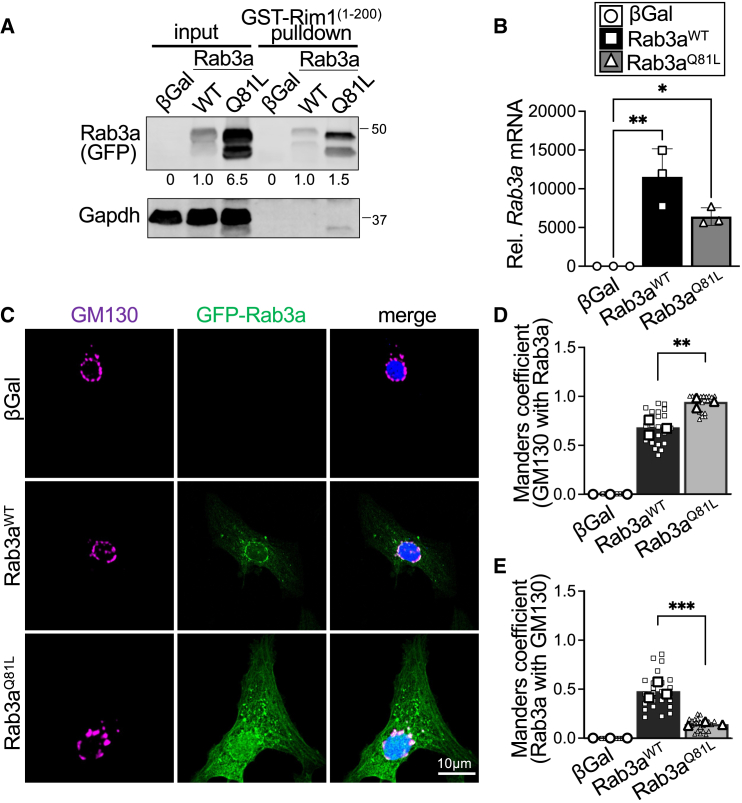
Constitutive Rab3a activity promotes Rab3a protein abundance and localization to the cell periphery. (*A*) Rab3-GTP pull-down assays followed by immunoblotting for input Rab3a protein levels and Rab3a-GTP levels (GST-Rim1^1−200^ pull-down) in neonatal rat cardiomyocytes (NRCMs) transduced with adenovirus to express GFP-tagged wild-type Rab3a (Rab3a^WT^) or constitutively active GTP-bound Rab3a (Rab3a^Q81L^), or β-galactosidase control (βGal). The higher-molecular-weight anti-GFP immunoreactive band migrating around 50 kDa was used for quantification of GFP-Rab3a, although the lower-molecular-weight band reliably shows a similar relative pattern. Total Rab3a levels were normalized to Gapdh and Rab3a-GTP levels were normalized to input Rab3a levels. Representative immunoblots are shown from *n* = 3 independent NRCM isolations. Also see Fig. S3. (*B*) *Rab3a* mRNA levels in NRCMs transduced with the indicated adenoviruses. Values represent mean ± standard deviation. (*C*) Representative images of NRCMs transduced with adenoviruses for GFP-Rab3a^WT^, GFP-Rab3a^Q81L^, or βGal control and immunostained for endogenous GM130 (magenta). Nuclei were stained blue with DAPI. Scale bar, 10 μm. Quantification of Manders correlation coefficients for (*D*) the fraction of the Golgi marker GM130 colocalized with GFP-Rab3a and (*E*) the fraction of GFP-Rab3a colocalized with GM130. *n* = 3 independent experiments with 70–100 cells analyzed for each experiment. Individual data points for Manders coefficients obtained from each image are shown, and the mean value for each independent experiment used for statistical testing is depicted by the larger symbols. ^∗^*p* < 0.05, ^∗∗^*p* < 0.01, ^∗∗∗^*p* < 0.001, one-way ANOVA with Tukey’s multiple-comparisons test.

To further investigate the functional relevance of Rab3a activation on ANP release, we transduced NRCMs with adenoviruses that express wild-type Rab3a (Rab3a^WT^), a constitutively active GTP-loaded Rab3a mutant (Rab3a^Q81L^), or βGal as a control and subjected lysates to Rab3-GTP pull-down assays to determine Rab3a activity. NRCMs expressing Rab3a^Q81L^ substantially augmented total levels of Rab3a compared to Rab3a^WT^ (Figs. 4 *A* and S3), achieving protein levels similar to cardiomyocytes with adenoviral-mediated expression Rab3a^WT^ and treated with PE (Figs. 1 *A*, 3 *A*, S1, and S2). As expected, Rab3a-GTP levels were elevated in Rab3a^Q81L^-transduced cardiomyocytes in comparison to cells that express wild-type Rab3a, even when normalized to respective total Rab3a protein levels (Figs. 4 *A* and S3). Notably, Rab3a^Q81L^-transduced NRCMs achieved similar relative steady-state levels of Rab3a-GTP to Rab3a^WT^-transduced NRCMs with activation of Rab3a in response to the G*⍺*q-coupled receptor agonist PE (Figs. 1 *A*, 3 *A*, and S1–S3). qPCR analyses showed similar mRNA expression of *Rab3a* in Rab3a^WT^- and Rab3a^Q81L^-transduced myocytes (Fig. 4 *B*), indicating differences in Rab3a protein levels and activity occur via a post-transcriptional mechanism and not due to alterations in viral titer or transduction efficiency. Immunofluorescent staining and imaging revealed expression of Rab3a^Q81L^ resulted in enhanced Rab3a protein levels and localization at the cardiomyocyte Golgi and as well as a greater portion of Rab3a protein at peripheral post-Golgi vesicles and endomembranes (GM130-negative) compared to cells expressing Rab3a^WT^ (Fig. 4 *C*–*E*), very similar to what is observed with PE treatment (Fig. 1 *B*–*D*), suggesting a role for active Rab3a in cardiomyocyte secretion.

**Figure 5:**
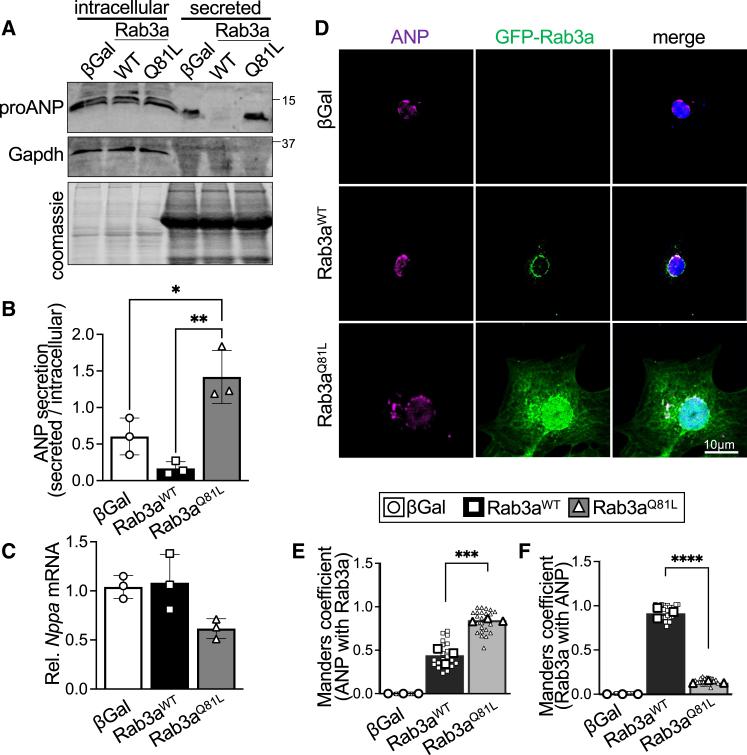
Rab3a activity is sufficient to elicit ANP secretion by cardiomyocytes. (*A*) Representative immunoblotting of intracellular and secreted ANP protein in culture medium from NRCMs transduced with adenoviruses for GFP-tagged wild-type Rab3a (Rab3a^WT^) or constitutively active GTP-bound Rab3a (Rab3a^Q81L^), or βGal control. The protein gel was Coomassie stained as a loading control for secreted protein and Gapdh immunoblotting was used as loading control for intracellular protein. *n* = 3 independent experiments. Quantification of (*B*) the ratio of secreted:intracellular proANP and (*C*) *Nppa* (ANP) mRNA levels in NRCMs transduced with adenoviruses for GFP-Rab3a^WT^, GFP-Rab3a^Q81L^, or βGal control. Values depicted in histograms are mean ± standard deviation. (*D*) Representative images of NRCMs transduced with adenoviruses for GFP-tagged Rab3a^WT^ or Rab3a^Q81L^, or βGal control and immunostained for endogenous ANP (magenta). Nuclei were stained blue with DAPI. Scale bar, 10 μm. Quantification of Manders coefficients for (*E*) the fraction of endogenous ANP colocalized with GFP-Rab3a and (*F*) the fraction of GFP-Rab3a colocalized with endogenous ANP. *n* = 3 independent experiments with 70–100 cells analyzed for each experiment. Individual data points for Manders coefficients obtained from each image are shown, and the mean value for each independent experiment used for statistical testing is depicted by the larger symbols. ^∗^*p* < 0.05, ^∗∗^*p* < 0.01, ^∗∗∗^*p* < 0.001, ^∗∗∗∗^*p* < 0.001, one-way ANOVA with Tukey’s multiple-comparisons test.

To directly assess regulation of cardiomyocyte ANP secretion by Rab3a activity, we performed immunoblotting assays on culture medium and cell lysates from NRCMs transduced with βGal, Rab3a^WT^, or Rab3a^Q81L^ to evaluate levels of secreted and intracellular ANP. Immunoblotting showed intracellular ANP protein levels were unchanged by overexpression of wild-type or constitutively active Rab3a (Fig. 5 *A*). However, expression of the permanently active Rab3a^Q81L^ mutant promoted ANP release from cardiomyocytes in comparison to cells that were transduced with Rab3a^WT^ or βGal (Fig. 5 *A*). Notably, secretion assays revealed that expression of Rab3a^WT^ if anything reduced secretion of ANP by cardiomyocytes when compared to cardiomyocytes expressing βGal control or Rab3a^Q81L^ (Fig. 5 *A* and *B*). qPCR analyses demonstrated *Nppa* mRNA levels were unaltered by expression of Rab3a^WT^ or Rab3a^Q81L^ (Fig. 5 *C*), suggesting effects of Rab3a overexpression on ANP secretion occur post-transcriptionally. Intracellular ANP levels were not altered by expression of Rab3a^WT^ or Rab3a^Q81L^ as assessed by confocal imaging (Fig. 5 *D*), consistent with immunoblotting results (Fig. 5 *A*), indicating Rab3a activity can promote ANP secretion independent of effects on ANP biosynthesis. ANP was localized in a punctate, vesicular pattern predominantly in the perinuclear region consistent with the Golgi in βGal- and Rab3a^WT^-transduced cells, whereas expression of the constitutively active Rab3a^Q81L^ mutant resulted primarily in a very similar punctate, vesicular pattern of ANP in the perinuclear region that was accompanied by an increase in ANP puncta at the cell periphery (Fig. 5 *D*) and portion of ANP colocalized with Rab3a (Fig. 5 *D* and *E*). The substantial increase in Rab3a protein levels and broader intracellular distribution of the Rab3a^Q81L^ mutant compared to Rab3a^WT^ in unstimulated NRCMs resulted in a reduction in the fraction of Rab3a colocalized with ANP (Fig. 5 *D* and *F*), similar to PE-treated NRCMs expressing Rab3a^WT^ (Fig. 2 *D* and *F*). Notably, genetic activation of Rab3a by expression of Rab3a^Q81L^ recapitulates the increased Rab3a protein abundance, activity, and intracellular localization observed with Rab3a^WT^ in response to PE (Figs. 1 *A*–*D*, 2 *D*–*F*, 3 *A*, 4 *A*, *C*–*E*, and 5 *A*–*F*) and similarly stimulates ANP secretion by NRCMs (Fig. 2 *A*, *B*, 3 *B*, *C*, 5 *A*, and *B*). Collectively, these data suggest GTP-loaded Rab3a may promote expansion of and/or preferentially localize to post-Golgi vesicles in cardiomyocytes to facilitate exocytosis and ANP secretion, similar to its canonically described roles in neuromodulator and neurotransmitter release (5,20).

## Discussion

Cardiomyocytes secrete natriuretic peptides into the circulation in response to cardiovascular stress as an adaptive mechanism to lower blood pressure and cardiac workload through activation of the guanylate cyclase-coupled NPR-A receptors (1,7,10). Although natriuretic peptide release by atria in response to stretch is primarily mediated by signaling through the heterotrimeric G protein *⍺* subunit, G*⍺*o (35,36), it is also robustly enhanced in response to activation of G*⍺*q-coupled receptors such as activation of AT1R by elevated circulating angiotensin-II in cardiovascular disease (1,2,37,38). Despite the clinical relevance, mechanisms underlying anterograde trafficking of natriuretic peptide-containing secretory vesicles through the endomembrane system to elicit release of natriuretic peptides into the circulation remain largely unknown. We previously reported that PE, which activates the G*⍺*q-coupled *⍺*1-adrenergic receptor, induces both total protein levels and GTP loading of Rab3a in cardiomyocytes (3). Here, we found PE-induced activation of Rab3a and secretion of ANP by cardiomyocytes are G*⍺*q dependent. Pharmacological inhibition of G*⍺*q impaired activation of Rab3a in response to PE, suggesting a guanine nucleotide exchange factor (GEF) for Rab3a could be activated downstream of G*⍺*q. G*⍺*q mediates activation of Rho family small GTPases such as Rac1 and RhoA to control actin cytoskeletal dynamics, cell migration, and proliferation through direct interaction with and activation of the RhoGEFs, Trio (39,40) and p63RhoGEF (40,41). Here we found a novel function for G*⍺*q in mediating activation of the small GTPase Rab3a in cardiomyocytes downstream of receptor activation that is required for effective exocytosis and ANP release, although the precise mechanism of Rab3a activation and identity of the Rab3 GEF in cardiomyocytes remain to be elucidated.

Rab proteins are the largest family of small GTPases, consisting of more than 60 isoforms, and coordinate membrane fusion events to modulate intracellular trafficking, membrane dynamics, and secretory pathway activity (42,43). Rab3 protein isoforms (Rab3A/B/C/D) have more specific roles in aiding tethering, docking, and/or fusion of secretory vesicles with the plasma membrane for exocytic release of neurotransmitters, neuromodulators, and peptide hormones (3,4,5,20,44). G*⍺*q plays critical roles in promoting exocytosis and secretion in pancreatic *β* cells, platelets, and neurons indirectly through canonical IP3-dependent mobilization of intracellular calcium stores and G*βγ*-mediated modulation of ion channel current, membrane potential, and consequent promotion of calcium influx necessary for activation of membrane fusion machinery (45,46,47,48). We found impairment of ANP release by inhibiting G*⍺*q was associated with a deficit in PE-stimulated activation of Rab3a that is required for efficient ANP secretion in cardiomyocytes (3), implicating a role for G*⍺*q in more proximally stimulating secretory pathway flux through promotion of the Rab3 GTPase cycle in cardiomyocytes. Whether G*⍺*q directly activates a GEF for Rab3a in cardiomyocytes or facilitates receptor-dependent Rab3a activation by a more indirect mechanism remains to be elucidated. Nonetheless, G*⍺*q could coordinately promote ANP release in cardiomyocytes by both enhancing Rab3 GTPase activity as well as through activation of calcium-dependent exocytic membrane fusion machinery. However, inhibition of G*⍺*q markedly reduced intracellular and secreted levels of ANP both at baseline and response to PE, suggesting G*⍺*q signaling is not only required for stress-inducible induction of Rab3a activity to promote secretory pathway activity and exocytosis of ANP but also contributes to basal ANP synthesis, production, and/or secretion.

Downstream of G*⍺*q or other activating stimuli, Rab3 functions as an indispensable regulatory component of the exocytic membrane fusion machinery through specific interaction of Rab3-GTP on the vesicle membrane with effectors such as RIM1 and Munc13 that facilitate interaction with plasma membrane phospholipids and t-SNAREs to promote priming, docking, and ultimate fusion of secretory vesicles with the plasma membrane that results in extracellular release of their contents (4,5,6,19,20,44). Here, we found Rab3a activation was required for effective cardiomyocyte exocytosis and ANP release. Not only did inhibition of G*⍺*q impair PE-stimulated ANP release that was associated with a deficit in activation and localization of Rab3a to post-Golgi vesicular membranes but expression of a constitutively active (GTP-loaded) Rab3a mutant was sufficient to robustly induce ANP secretion in cardiomyocytes, essentially phenocopying induction of Rab3-mediated exocytosis and ANP release observed in response to a G*⍺*q-coupled receptor agonist. Sequential cycles of GEF-dependent GTP loading of Rab3, potentially downstream of G*⍺*q, followed by GAP-dependent inactivation of Rab3, dissociation of Rab3 from the membrane, and chaperoning back to the Golgi for reinitiation of the cycle and successive rounds of secretory vesicle fusion with the plasma membrane are likely required for release of physiologically relevant levels of natriuretic peptides, neuromodulators, neurotransmitters, and other hormones (6,49). Indeed, overexpression of Rab3gap1, which mediates GTP hydrolysis on Rab3 resulting in its inactivation and dissociation from the vesicle/plasma membrane (6,49,50,51), provokes robust secretion of ANP by cardiomyocytes whereas a GAP-deficient point mutant (3) or palmitoylation-deficient mutant with mislocalized GAP activity (52) lack this effect, suggesting the Rab3 GTPase cycle is a necessary and fundamental component of exocytic natriuretic peptide release by cardiac myocytes.

We found the Rab3 GTPase cycle to be essential for exocytosis and ANP secretion by cardiomyocytes but the findings herein are also likely applicable to Rab3-mediated regulation of BDNF and neurotransmitter release by neurons (5,51), insulin secretion by pancreatic *β* cells (53,54), and catecholamine release by chromaffin cells (55,56). The constitutively GTP-loaded Rab3a^Q81L^ mutant markedly induced ANP release by cardiomyocytes, and although once loaded with GTP this Rab3a^Q81L^ mutant is incapable of inactivation for further rounds of the Rab3 GTPase cycle, it is possible that high levels of exogenously expressed, stably GTP-loaded Rab3a^Q81L^ are sufficient to promote exocytosis and ANP release in cardiomyocytes through superphysiologic association with secretory vesicles and Rab3 effectors that would otherwise require highly regulated modulation of the Rab3 GTPase cycle by GEFs, GAPs, and other regulatory factors. It is also possible that the trend toward reduced ANP release observed with overexpression of wild-type Rab3a is due to an absence of stoichiometric amounts of GEFs and GAPs to promote GTPase cycling resulting in an overabundance of inactive Rab3a that could have a dominant-negative effect on exocytic release of ANP.

### Study limitations

Here, we dissected the molecular mechanisms governing exocytosis and ANP release utilizing NRCMs, a robust *in vitro* primary cell system readily amenable to genetic manipulation of Rab3 GTPase activity and assessment of release of endogenous proANP into the conditioned medium. These data suggest acceleration of the Rab3 cycle may increase ANP secretion and could potentially be harnessed to increase circulating levels of natriuretic peptides for cardiovascular protection in patients with hypertension and/or heart failure but need to be interpreted with caution. NRCMs have a constitutive secretory pathway that evokes sustained elevation in the synthesis, processing, and secretion of ANP in response to secretagogues such as PE (3,57). Regulation of exocytosis and ANP secretion in particular needs to be further examined in adult atria, which are the predominant source of natriuretic peptide production and release by the stressed heart *in vivo* (1,7,58). Atrial cardiomyocytes exhibit a secretory-contractile phenotype with a more expansive endomembrane system compared to ventricular cardiomyocytes and contain specific atrial granules, specialized secretory vesicles containing processed pronatriuretic peptides poised for stimulated release, akin to dense core vesicles that mediate exocytosis and endocrine signaling by neurons, adrenal chromaffin cells, and pancreatic *β* cells (1,57,59,60,61). Unfortunately, there also are not established reliable markers for secretory vesicles and endomembrane compartments in NRCMs, preventing in-depth assessment of the identity of ANP-containing vesicles and intracellular location of Rab3 activation.

In studies in NRCMs herein we observed repression of intracellular and secreted ANP protein levels in unstimulated cells when inhibiting G*⍺*q as well as repression of the PE-stimulated induction of *Nppa* mRNA levels that certainly contribute to the impairment of PE-stimulated ANP release, and thus we were unable to completely dissociate the antihypertrophic effects and repression of ANP biosynthesis by G*⍺*q inhibition from its impacts on Rab3a activation and ANP secretion. Notably, inhibition of G*⍺*q in NRCMs acutely prevented GTP loading of Rab3a and ANP secretion in response to 3 h of PE treatment, before transcriptional changes, and other lines of evidence suggest this enhancement of Rab3a activity could play a role in NRCM exocytosis and ANP secretion downstream of G*⍺*q-coupled receptors. Similar to PE stimulation, expression of a constitutively active Rab3a mutant was sufficient to stimulate secretion of endogenous ANP by NRCMs without altering *Nppa* mRNA levels. Additionally, genetic manipulation of the Rab3 cycle in NRCMs by adenoviral expression of a Rab3gap1 palmitoylation-deficient mutant with spatially restricted GAP activity mitigated the ability of Rab3gap1 overexpression to promote basal and PE-stimulated secretion of endogenous ANP without altering intracellular ANP protein levels (52). Thus, although NRCMs have a constitutive secretory pathway and are not ideal for modeling secretion by the adult heart, these studies demonstrate a role for Rab3a in cardiomyocyte exocytosis and a substantial effect on ANP secretion. Notwithstanding, Rab3a activity and G*⍺*q-dependent activation of Rab3a could potentially play an even more prominent role in stimulated ANP release through a regulated secretory pathway by atria *in vivo* where Rab3a localizes to atrial granules (3), which are the major source of natriuretic peptides released into the circulation in response to cardiovascular stress (1,7,58).

## Acknowledgments

This work was supported by grants from the 10.13039/100000002National Institutes of Health (R01HL166274 to M.J.B.) and the 10.13039/100000968American Heart Association (827440 to K.E.).

## Author contributions

K.E. and M.J.B. conceptualized and designed the studies. K.E., A.S., and S.K. performed experiments. K.E. and M.J.B. wrote the manuscript.

## Declaration of interests

The authors declare no competing interest.

## Supporting material

Document S1. Figures S1–S3

Document S2. Article plus supporting material
