# Supplementary material for: The Rab3 GTPase cycle modulates cardiomyocyte exocytosis and atrial natriuretic peptide release
Source: Biophys J. 2025 Mar 20;124(11):1856–66. doi: 10.1016/j.bpj.2025.03.013 (PMC12256856; doi:10.1016/j.bpj.2025.03.013)
Supplement: Document S1. Figures S1–S3 [file mmc1.pdf]

**Biophysical Journal, Volume *124***

## Supplemental information

# The Rab3 GTPase cycle modulates cardiomyocyte exocytosis and atrial natriuretic peptide release

**Kobina Essandoh, Arasakumar Subramani, Sribharat Koripella, and Matthew J. Brody**

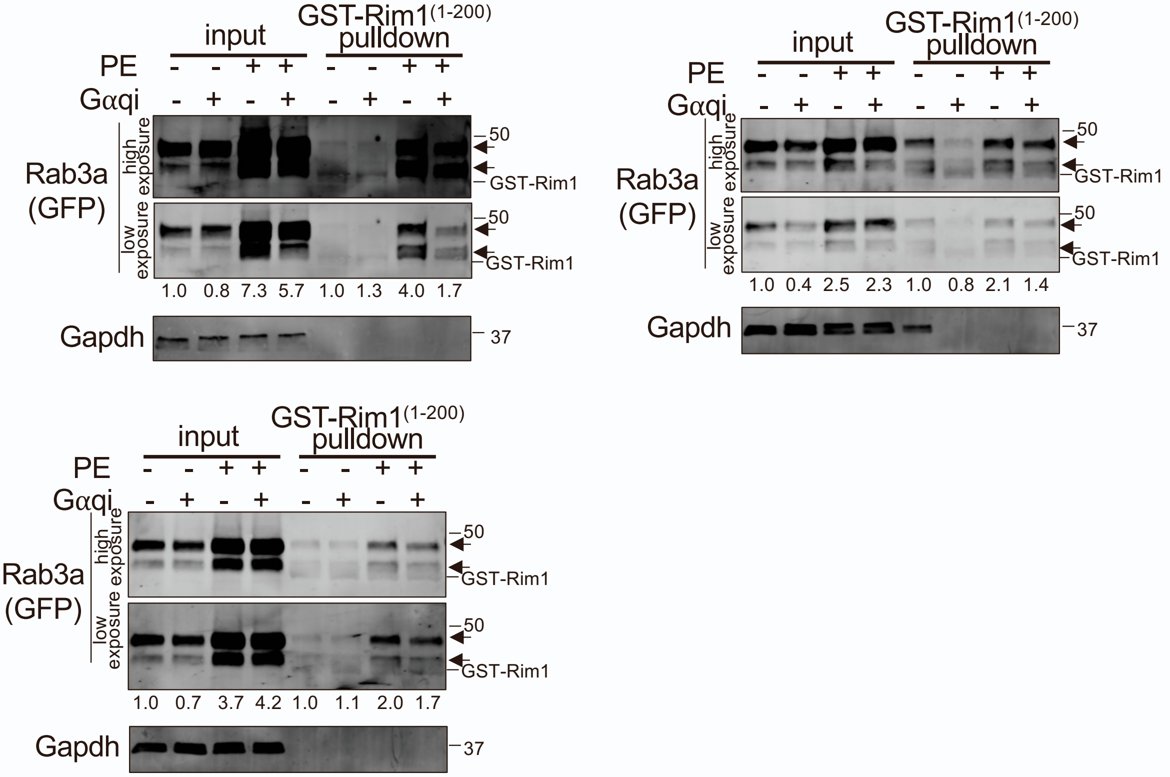


**Figure S1.** Gαq-dependent activation of Rab3a in cardiomyocytes. Related to Fig. 1 *A*. Additional biological replicates of Rab3a-GTP pulldown assays followed by immunoblotting for input Rab3a protein levels and Rab3a-GTP levels (GST-Rim1$^{1-200}$ pulldown) in neonatal rat cardiomyocytes (NRCMs) transduced with adenovirus to express GFP-Rab3a and treated with PBS or phenylephrine (PE) for 24 hours with or without FR900359 (Gαqi, 1 μM). The higher molecular weight anti-GFP immunoreactive band migrating around 50 KDa was used for quantification of GFP-Rab3a although the lower molecular weight band reliably shows a similar relative pattern. Total input GFP-Rab3a protein was normalized to Gapdh levels and GFP-Rab3a-GTP levels were normalized to total GFP-Rab3a protein levels in the input.

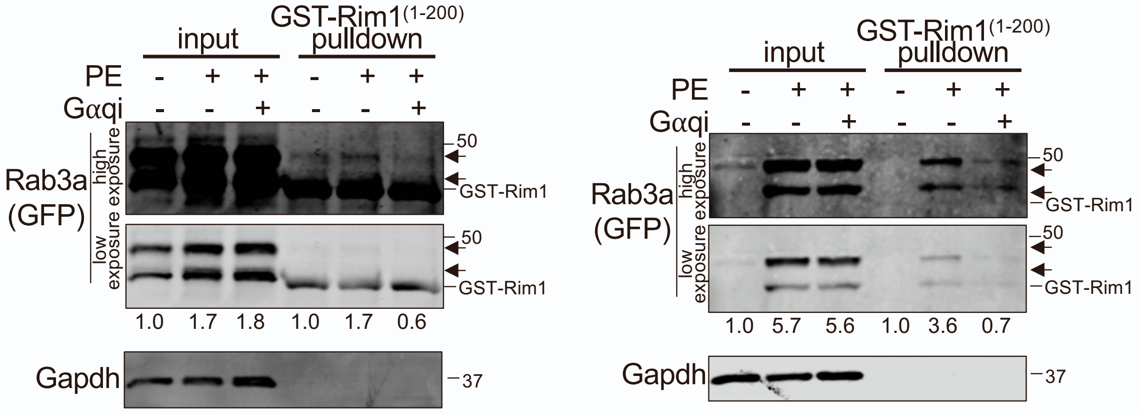


**Figure S2.** Acute Gαq-dependent activation of Rab3a in cardiomyocytes. Related to Fig. 3 *A*. Additional biological replicates of Rab3a-GTP pulldown assays followed by immunoblotting for input Rab3a protein levels and Rab3a-GTP levels (GST-Rim1$^{1\text{-}200}$ pulldown) in neonatal rat cardiomyocytes (NRCMs) transduced with adenovirus to express GFP-Rab3a and treated with PBS or phenylephrine (PE) for 3 hours with or without FR900359 (Gαqi, 1 μM). The higher molecular weight anti-GFP immunoreactive band migrating around 50 KDa was used for quantification of GFP-Rab3a although the lower molecular weight band reliably shows a similar relative pattern. Total input GFP-Rab3a protein was normalized to Gapdh levels and GFP-Rab3a-GTP levels were normalized to total GFP-Rab3a protein levels in the input.

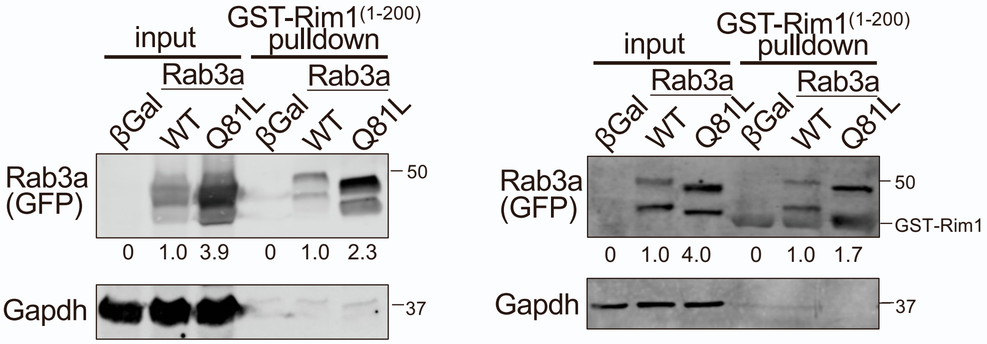


**Figure S3.** Rab3a activity in cardiomyocytes expressing the constitutively-active GTP-bound Rab3a. Related to Fig. 4 *A*. Additional biological replicates of Rab3a-GTP pulldown assays followed by immunoblotting for input Rab3a protein levels and Rab3a-GTP levels (GST-Rim1$^{1\text{-}200}$ pulldown) in neonatal rat cardiomyocytes (NRCMs) transduced with adenovirus to express GFP-tagged wildtype Rab3a (Rab3a$^{WT}$) or constitutively-active GTP-bound Rab3a (Rab3a$^{Q81L}$), or β-galactosidase control (βGal). The higher molecular weight anti-GFP immunoreactive band migrating around 50 KDa was used for quantification of GFP-Rab3a although the lower molecular weight band reliably shows a similar relative pattern. Total Rab3a levels were normalized to Gapdh and Rab3a-GTP levels were normalized to input Rab3a levels.
